# Complaints of reduced cognitive functioning during perimenopause: a cross-sectional analysis of the Japan Nurses’ Health Study

**DOI:** 10.1186/s40695-022-00076-9

**Published:** 2022-06-05

**Authors:** Kunihiko Hayashi, Yuki Ideno, Kazue Nagai, Jung-Su Lee, Toshiyuki Yasui, Takumi Kurabayashi, Kiyoshi Takamatsu

**Affiliations:** 1 Graduate School of Health Sciences, Gunma University, 3-39-22 Showa-machi, Maebashi, Gunma 371-8514 Japan; 2 Center for Mathematics and Data Science, Gunma University, Maebashi, Japan; 3 Postgraduate School of Healthcare, Tokyo Healthcare University, Tokyo, Japan; 4 Institute of Biomedical Sciences, Tokushima University Graduate School, Tokushima, Japan; 5 Department of Obstetrics & Gynecology, Niigata City General Hospital, Niigata, Japan; 6 Department of Obstetrics and Gynecology, Tokyo Dental College Ichikawa General Hospital, Chiba, Japan

**Keywords:** Menopausal symptoms, Poor memory and forgetfulness, Complaint of reduced cognitive functioning, Somatic symptoms, Psychological symptoms, Vasomotor symptoms

## Abstract

**Background:**

Many women experience various symptoms during the period of menopausal transition, including complaints of reduced cognitive functioning. However, these complaints are not necessarily recognized as core menopausal symptoms. In this study, we sought to characterize subjective complaints of reduced cognitive functioning by analyzing cross-sectional data from the Japan Nurses’ Health Study (JNHS).

**Methods:**

The JNHS 4-year follow-up questionnaire containing a 21-item climacteric symptom checklist, which included a question about “poor memory or forgetfulness”, was mailed between 2005 and 2011 to all JNHS participants, regardless of their age at the time of the survey. We estimated the prevalence of slight and severe complaints in 5-year age-groups. We used principal component analysis to explore the underlying factors among the 21 symptoms during the menopausal transition period in women aged 45–54 years at the time of the survey. We also examined risk factors for complaints using multivariable modified Poisson regression analysis.

**Results:**

In total, 12,507 women responded to the 4-year survey. The mean age at the time of the 4-year survey was 46.5 years (range 27–82). “Poor memory or forgetfulness” showed a peak prevalence of 81.7% (severe 27.9%; slight 53.8%) at 50–54 years, and gradually decreased after 55 years. Principal component analysis indicated that “poor memory or forgetfulness” belonged to somatic symptoms and was close to psychological symptoms in women aged 45–54 years. In women aged 45–54 years, the complaint was also significantly associated with hot flashes and sweats. Multivariable modified Poisson regression analysis showed that menopausal status (uncertain and postmenopausal), less sleep (sleep of < 5 h and sleep of 5– < 6 h), night-shift work, and severe vasomotor symptoms (VMS) were significantly associated with the prevalence of severe complaints of reduced cognitive functioning in women aged 45–54 years.

**Conclusions:**

We found that prevalence of “poor memory or forgetfulness” was highest during the menopausal transition period and among perimenopausal women. This subjective complaint was associated with somatic, psychological complaints and VMS. It may be useful for women with cognitive problems in the transition period to consider management of comorbid menopausal symptoms.

## Background

During the period of menopausal transition, many women experience various symptoms and complaints, including reduced cognitive functioning. These menopausal symptoms can be burdensome and reduce quality of life. Many questionnaires and checklists have been created to assess menopausal symptoms. Some questionnaires addressing menopausal symptoms include items related to reduced cognitive functioning. For example, the Women’s Health Questionnaire includes the item “My memory is poor” [1]. Within the item concerning physical and mental exhaustion, the Menopausal Symptom List includes descriptions of two complaints: impaired memory and forgetfulness [2]. The Climacteric Symptom Checklist for Japanese Women includes an item related to poor memory: “poor memory (difficulty remembering things) or forgetfulness (often forgetful)” [3].

Reduced cognitive functioning is one of the most prevalent complaints during the menopausal transition period and was found to be the third-most frequent complaint among Japanese women presenting at the gynecologic outpatient clinics of university hospitals [3]. Using the self-administered Memory Functioning Questionnaire in the Seattle Midlife Women’s Health Study, 72% of women in the midlife age range reported difficulty remembering names [4]. Previous research has indicated a significant role of estrogen in cognitive functioning, and it has been hypothesized that menopausal hormone replacement therapy (HRT) could enhance cognition if administered at menopause [5, 6]. However, frequently used questionnaires dealing with menopausal symptoms do not always include items concerning reduced cognitive functioning [7–9]. Thus, reduced cognitive functioning is not necessarily recognized as a core menopausal symptom.

It is currently unclear whether subjective complaints of reduced cognitive functioning are related to menopausal transition or advancing age. Controversy currently exists regarding whether there is a significant decline in cognitive functioning during menopausal transition and subsequent improvement in such functioning after that period, or whether the decline is not specific to menopausal transition [10]. To date, complaints of reduced cognitive functioning at each stage of women’s lives have not been well documented. Moreover, it is currently unclear how complaints of reduced cognitive functioning correlate with other climacteric symptoms, such as vasomotor symptoms (VMS). As the first step in answering these questions, data regarding various subjective symptoms in women across a wide age range are needed to characterize the cognitive complaints during menopausal transition.

In the present study, we conducted cross-sectional analyses of follow-up survey data in a prospective women’s cohort study: the Japan Nurses’ Health Study (JNHS). We sought to characterize the complaints of reduced cognitive functioning by analyzing cross-sectional data regarding various subjective symptoms in women across a wide age range.

## Methods

### Participants

The JNHS is a nationwide prospective cohort study designed to investigate the effects of lifestyle and health-care practices on the health of Japanese women during their life course. Participants were recruited from all 47 prefectures in Japan from 2001 to 2007. In total, 15,019 female nurses who were registered nurses, licensed practical nurses, public health nurses, and/or midwives agreed to be followed up. At the time of enrollment, all participants provided written informed consent for the follow-up surveys. The cohort received biennial follow-up questionnaires by mail. The details of the study design have previously been reported [11].

From 2005 to 2011, 4 years after each participant’s enrollment, a 4-year follow-up questionnaire was mailed to participants. The self-administered questionnaire included the Climacteric Symptom Checklist for Japanese Women [3]. We asked all participants to complete the 21-item symptom checklist regardless of age and menopausal status.

### Climacteric Symptom Checklist

The Climacteric Symptom Checklist for Japanese Women was developed by a subcommittee of the Japan Society of Obstetrics and Gynecology. The checklist comprises 21 items: 1) hot flashes; 2) sweats; 3) difficulty falling asleep; 4) insomnia (difficulty remaining asleep); 5) excitability or irritability; 6) anxiety; 7) nervousness; 8) depressive mood; 9) fatigue or loss of volition; 10) eye strain; 11) poor memory (difficulty remembering things) or forgetfulness (often forgetful); 12) dizziness or vertigo; 13) palpitation; 14) chest tightness; 15) headaches; 16) neck or shoulder stiffness; 17) back pain; 18) joint pain in limbs; 19) cold feeling in waist or limbs; 20) numb feeling in limbs; and 21) over-sensitivity to sound [3]. Participants responded to the questions by selecting one of three response choices: none, slight, or severe.

### Demographic data and covariates

The demographic data and covariates included in the analyses were obtained from the self-administered baseline and 4-year follow-up questionnaires. These variables were as follows: age at 4-year survey, nurse license (registered nurse, licensed practical nurse, public health nurse, or midwife), educational degree (university and above or other), parity (0, 1, 2, or ≥ 3), marital status (single, married, or divorced or widowed), menopausal status (premenopausal, uncertain, or postmenopausal), body mass index (BMI) (< 18.5, 18.5- < 25.0, 25.0- < 30.0, or ≥ 30) calculated from self-reported height and weight, smoking status (current smoker or non-smoker), alcohol intake (none, once or twice a week, 3–6 times a week, or every day), sleeping hours (< 5, 5– < 6, 6– < 7, 7– < 8, or ≥ 8 h), night-shift work (no or yes), and current use of HRT (no or yes). We defined “postmenopausal” as the permanent cessation of menstrual periods or more than 1 year since the last menstruation. We included women who had menstrual bleeding because of HRT use in the postmenopausal category. Women who had undergone hysterectomy and whose age at the survey was 54 years or more were considered to be postmenopausal, because age at menopause was estimated to be ≤ 54 years in 90% of women who had undergone natural menopause in the JNHS population [12, 13]. We defined “uncertain” as participants who were unsure whether their menstrual periods had permanently ceased.

### Statistical analysis

Prevalence of women with a complaint of “poor memory or forgetfulness” in 5-year age-groups (< 34, 35–39, 40–44, 45–49, 50–54, 55–59, 60–64, and ≥ 65 years) was estimated to identify the group with peak prevalence.

The Climacteric Symptom Checklist includes 21 items, because women experience various complaints and symptoms in the climacteric period. We examined component structure among these correlated climacteric symptoms and the characteristics of severe complaints of “poor memory or forgetfulness” in the identified component structure. Principal component analysis was used to explore the underlying factors among the 21 symptoms in women aged 45–54 years, the age range defined as the climacteric period by the Japan Society of Obstetrics and Gynecology. Promax rotation was employed to allow for correlated factors. We retained factors with an eigenvalue greater than 1.0, and factor loadings of at least 0.4 were considered significant. Spearman’s rank correlation coefficients between the complaint of reduced cognitive functioning (none, slight, and severe) and other symptoms (none, slight, and severe) in the same extracted factors were calculated. The associations between severe complaints of reduced cognitive functioning and severe VMS, hot flashes and sweats, were examined using χ^2^ tests.

Multivariable modified Poisson regression analysis with the robust sandwich variance was used to examine the associations of demographic factors, lifestyle variables, BMI, reproductive history including menopausal status, severe VMS and current use of HRT with severe complaints of reduced cognitive functioning in women aged 45–54 years. The adjusted prevalence ratio (PR) and 95% confidence interval (95%CI) were calculated. We used SAS version 9.4 (SAS Institute Inc., Cary, NC, USA) for all data analyses. The statistical significance level was set at *P* = 0.05.

## Results

### Characteristics of the study population

Of the 15,019 participants who agreed to be followed up, 12,507 responded to the 4-year survey (response rate = 83.3%), leaving a study population of 12,226 women. Women who were pregnant at the time of the survey (*n* = 257) and individuals who did not fully answer the checklist questions (*n* = 24) were excluded. Characteristics of the study population are shown in Table 1. The mean age at the 4-year survey was 46.5 years (standard deviation, 8.1 years; range, 27–82 years). The proportion of women in the age range of menopausal transition (45–54 years) was 38.2%. In addition, 79.6% of the participants worked as registered nurses and 54.0% worked rotating night shift work.

**Table 1:** Participant characteristics (*n* = 12,226)

		n	%
Age at 4-year survey	< 34	545	4.5
	35–39	2,262	18.5
	40–44	2,503	20.5
	45–49	2,481	20.3
	50–54	2,187	17.9
	55–59	1,538	12.6
	60–64	585	4.8
	≥ 65	125	1.0
Nursing license	registered nurse	9,735	79.6
	licensed practical nurse	814	6.7
	public health nurse	619	5.1
	midwife	1,021	8.4
	n/a	37	0.3
Educational degree	university or above	486	4.0
	other	11,740	96.0
Parity	0	3,311	27.1
	1	1,535	12.6
	2	4,333	35.4
	≥ 3	2,742	22.4
	n/a	305	2.5
Marital status	single	2,382	19.5
	married	8,728	71.4
	divorced / widowed	991	8.1
	n/a	125	1.0
Menopausal status	premenopausal	7,838	64.1
	uncertain	556	4.6
	postmenopausal	3,830	31.3
	n/a	2	0.0
Body Mass Index	< 18.5	956	7.8
	18.5– < 25.0	9,325	76.3
	25.0– < 30.0	1,567	12.8
	≥ 30.0	281	2.3
	n/a	97	0.8
Smoking	current smoker	1,378	11.3
	non-smoker	10,836	88.6
	n/a	12	0.1
Alcohol intake	no	4,426	36.2
	once or twice a week	4,924	40.3
	3–6 times a week	1,576	12.9
	every day	1,262	10.3
	n/a	38	0.3
Sleeping hours	< 5 h	526	4.3
	5– < 6	4,073	33.3
	6– < 7	5,333	43.6
	7– < 8	1,861	15.2
	≥ 8	398	3.3
	n/a	35	0.3
Night-shift work	no	5,622	46.0
	yes	6,604	54.0
Current HRT use	no	11,581	94.7
	yes	596	4.9
	n/a	49	0.4

*n/a* not available (missing data)

*HRT* Hormone replacement therapy

### Subjective cognitive functioning by age-group

**Fig. 1:**
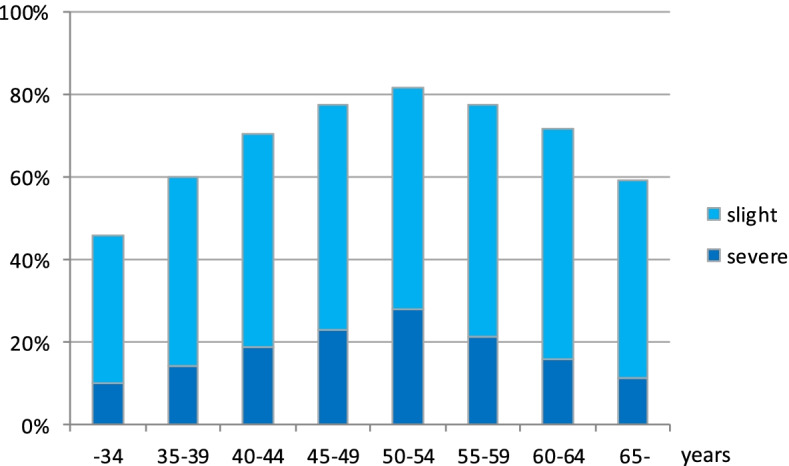
Prevalence of complaints of "poor memory and forgetfulness" by age-group (*n* = 12,226)

Figure 1 shows the prevalence of the subjective complaint of “poor memory or forgetfulness” in the Climacteric Symptom Checklist for Japanese Women in eight 5-year age-groups. The subjective complaint showed a peak prevalence of 81.7% (severe, 27.9%; slight, 53.8%) in the age-group of 50–54 years, then gradually decreased after 55 years. This was the most common complaint among the 21-item subjective symptoms in women aged 50–54 years.

### Principal component analysis in the age range of menopausal transition

The factors extracted by principal component analysis among the 21-item subjective symptoms during the midlife age range of menopausal transition (45–54 years, *n* = 4,668) are shown in Table 2. According to the criterion of an eigenvalue greater than 1.0, six factors were identified. The six factors accounted for 56.4% of common variance. The first factor extracted (factor 1) corresponded to psychological symptoms. Factor 2 corresponded to somatic complaints, factor 3 to autonomic complaints, factor 4 to sensory and musculoskeletal complaints, factor 5 to sleep disturbance, and factor 6 to VMS. The loading factors of “poor memory or forgetfulness” were 0.370, 0.469, 0.083, 0.082, 0.047, and 0.139 for factors 1, 2, 3, 4, 5, and 6, respectively. According to the criterion of a loading factor of at least 0.4, “poor memory or forgetfulness” belonged to the somatic symptoms group (factor 2) and was also close to the psychological symptoms group (factor 1).

**Table 2:**
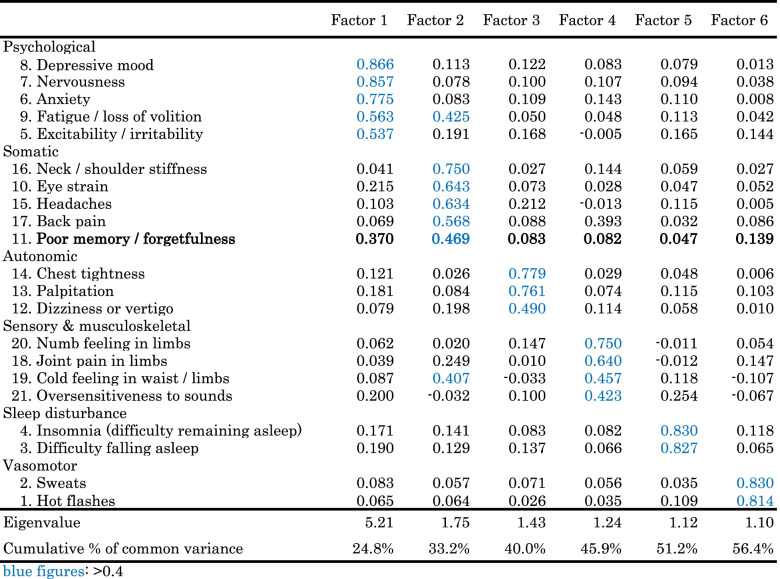
Rotated factor loadings extracted by principal component analysis

Spearman’s rank correlation coefficients for the complaint of reduced cognitive functioning were 0.391 with fatigue or loss of volition (factor 1), 0.333 with depressive mood (factor 1), 0.314 with nervousness (factor 1), 0.376 with eye strain (factor 2), 0.281 with excitability or irritability (factor 1), 0.235 with headaches (factor 2), 0.261 with back pain (factor 2), and 0.261 with neck or shoulder stiffness (factor 2).

### Multivariable modified Poisson regression analysis

**Fig. 2:**
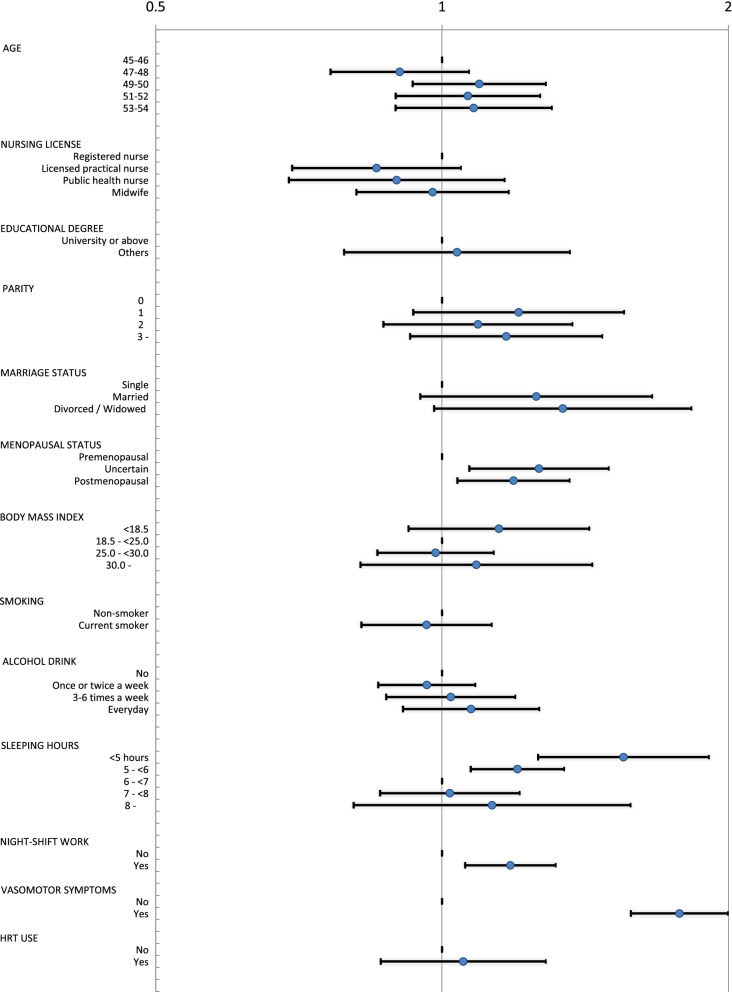
Multivariable adjusted prevalence ratios and 95% confidence intervals of severe complaints of reduced cognitive functioning for women aged 45–54 years

Multivariable modified Poisson regression analysis revealed that uncertain status (PR 1.26; 95%CI 1.07–1.50; vs premenopausal status), postmenopausal status (PR 1.19; 95%CI 1.04–1.36; vs premenopausal status), sleep of < 5 h (PR 1.55, 95%CI 1.26–1.91; vs 6– < 7 h), sleep of 5– < 6 h (PR 1.20, 95%CI 1.07–1.34; vs 6– < 7 h), night-shift work (PR 1.18; 95%CI 1.06–1.32; vs no night-shift work), and severe VMS (PR 1.78; 95%CI 1.58–2.00; vs no) were independently associated with the prevalence of severe complaints of reduced cognitive functioning (Fig. 2).

The association with VMS, one of the most burdensome climacteric symptoms, was examined. Severe complaints of reduced cognitive functioning were significantly associated with severe VMS, hot flashes (χ2 = 91.3, *P *< 0.001), and sweats (χ2 = 94.0, *P* < 0.001). The adjusted PR (95%CI) was 1.77 (1.53–2.04) for hot flashes and 1.67 (1.48–1.89) for sweats (Table 3).

**Table 3:** The association of severe complaints between conitive functioning and vasomotor symptoms

		Poor memory or forgetfulness							
		none + slight		severe		total	χ^2^ test	Adjusted Prevalence Ratio (95%CI) *	*P*-value
		n	%	n	%				
Hot flashes	none + slight	3310	76.5	1016	23.5	4326	χ^2^ = 91.3	1.77 (1.53–2.04)	< 0.0001
	severe	182	53.2	160	46.8	342	*P* < 0.0001		
Sweats	none + slight	3136	77.2	927	22.8	4063	χ^2^ = 94.0	1.67 (1.48–1.89)	< 0.0001
	severe	356	58.8	249	41.2	605	*P* < 0.0001		

^*^: adjusted for age, nursing license, educational degree, parity, marriage status, menopausal status, body mass index, smoking, alchohol drink, sleeping hours, night-shift work, and current HRT use

## Discussion

In the present study, approximately 80% of Japanese women in the period of menopausal transition reported a complaint of “poor memory or forgetfulness,” which was the most common complaint among the 21 menopausal symptoms. This high prevalence is supported by previous studies that reported that more than 70% of Japanese women in the midlife age range expressed complaints of frequent forgetfulness [3] and 72% of women in the midlife age range in the United States exhibited problems with remembering names [4]. The complaint of reduced cognitive functioning showed a peak prevalence in the age range of menopausal transition and gradually decreased after 55 years in the present study. We observed a similar trend for hot flashes and sweats (data not shown). Most previous cross-sectional studies have investigated symptoms and complaints only among women during the menopausal period. To the best of our knowledge, the present study is the first cross-sectional investigation to examine the prevalence of complaints among women in a wide age range (30 s–60 s) regardless of menopausal status. This finding is also supported by previous longitudinal studies that reported that cognitive test scores declined during the menopausal transition and appeared to attenuate the decline during the postmenopausal period [14, 15]. The age-independent decline during menopausal transition may be affected by both hormonal changes and other climacteric symptoms such as VMS.

In principal component analysis among the 21-item subjective symptoms during the period of menopausal transition, “poor memory or forgetfulness” presented a unique feature. The complaint of reduced cognitive functioning belonged to the somatic symptoms group and was very close to the psychological symptoms group. Specifically, this complaint was correlated with fatigue or loss of volition, depressive mood, nervousness, and eye strain. These findings support the inclusion of impaired memory and forgetfulness in the category of “Physical and mental exhaustion” in the Menopause Rating Scale [2]. Complaints of severely reduced cognitive functioning were significantly associated with severe VMS. Women with severe hot flashes or severe sweats exhibited approximately twice the prevalence of complaints of severely reduced cognitive functioning compared with women without severe hot flashes or severe sweats, with prevalence of 46.8% vs 23.5% for hot flashes and 41.2% vs 22.8% for sweats, respectively. The adjusted PR was statistically significant for hot flashes and sweats. The high prevalence of cognitive complaints and other somatic and psychological symptoms during menopausal transition may in part be related severe VMS.　Although subjective VMS is generally unrelated to memory performance, a higher frequency of physiological VMS using ambulatory monitors was reported to be associated with lower performance on a verbal memory test [16, 17]. The strong association between subjective VMS and complaints of reduced cognitive functioning observed in the present study might have occurred because the nurses were likely to self-report their subjective symptoms accurately as similar status of physical symptoms and/or because we examined the association by focusing on severe symptoms and severe complaints.

Multivariable modified Poisson regression analysis in individuals during the period of menopausal transition revealed that the prevalence of severe complaints of reduced cognitive functioning was significantly higher in women with uncertain status and who were postmenopausal, those with short sleeping hours, who worked night-shifts, and had severe VMS. Although short sleeping hours and night-shift work do not necessarily cause cognitive complaints, Hue et al. found a bidirectional relationship between sleep duration and cognitive function in a cohort study [18].The strong association with severe VMS suggests that the cognitive complaints during menopausal transition may in part be related to severe VMS. Current HRT use was not associated with complaints of reduced cognitive functioning in our study. However, we were unable to draw the conclusion that HRT has no effects because we did not identify the timing of HRT in current users. The Study of Women’s Health Across the Nation reported that HRT use in perimenopause was positively associated with cognitive performance, whereas the initiation of HRT after the final menstrual period was negatively associated [15]. Maki et al. reviewed previous literature and concluded that it is premature to make causal claims about VMS and memory dysfunction, but initial findings have raised the possibility that women with VMS might experience an improvement in cognition with VMS treatment [16].

This study involved several limitations that should be considered. First, our study population consisted of female nurses. Thus, the study population was likely to have been exposed to different risk and lifestyle factors compared with women in the general population. For example, short sleeping hours and night-shift work, which we identified as risk factors for severe complaints of cognitive functioning, may represent specific issues for nurses. However, an important benefit of this study population is that, owing to their medical knowledge and experience, nurses are likely to report information more accurately than the general population. We have no reason to suspect that the results with a general population of women would substantively differ from our findings, which characterized complaints during the menopausal transition period. Second, selection bias may be a concern regarding cognitive impairment. We ascertained information about climatic symptoms based on responses to a 4-year follow-up questionnaire. Non-responders to the mail survey may have included women who were unable to respond because of severe cognitive impairment. However, it is unlikely that middle-aged working nurses who had responded to the baseline questionnaire had undergone such a severe decline in 4 years. Third, this was a cross-sectional analysis, and we therefore could not completely separate the effects of age and menopause. The trajectory of the complaints within an individual, along with changing menopausal status, could not be described because of the nature of cross-sectional analyses. As a result of this limitation, we could not draw conclusions regarding the effect of HRT on complaints of reduced cognitive functioning. We plan to conduct prospective evaluations in a 14-year follow-up survey using a follow-up questionnaire including the same 21-item climacteric symptom checklist and confirm the findings of the present study by adding more information after age 65 in the near future.

Complaints related to cognitive functioning were associated with nonspecific somatic symptoms (e.g., eye strain), psychological symptoms (e.g., fatigue or loss of volition, depressive mood, and nervousness) and VMS. Some of the subjective complaints regarding cognitive functioning in the menopausal transition period might diminish with improved somatic and psychological symptoms and VMS. Thus, women with subjective complaints in the menopausal transition period and their health-care providers should consider management of comorbid menopausal symptoms, such as fatigue or loss of volition, depressive mood, and VMS.

## Conclusions

We found that the prevalence of complaints of poor memory and forgetfulness were highest during the period of menopausal transition and among perimenopausal women. Subjective complaints regarding cognitive functioning were associated with somatic and psychological complaints and VMS. Women with this problem during the menopausal transition period and their health-care providers should consider management of comorbid menopausal symptoms. Further research involving longitudinal studies will be needed to clarify the associations among subjective complaints during menopausal transition, VMS, and HRT use.

## Data Availability

The tabulated data that support the findings of this study are available from the corresponding author (KH) upon reasonable request. The raw data are not publicly available because providing these data was not included in the informed consent.

## Abbreviations

JNHS: Japan Nurses’ Health Study
VMS: Vasomotor symptoms
HRT: Hormone replacement therapy
BMI: Body mass index
PR: Prevalence ratio
95%CI: 95% Confidence interval
